# Case of Acute Bilious Fever, Successfully Treated by Nitric Acid

**Published:** 1808-02

**Authors:** Isaac Auld

**Affiliations:** Edisto, South Carolina


*Case of acute Bilious Fever, successfully treated by Nitric Acid. By* Isaac Auld, M. D. *of Edisto, South Carolina. Read before the Medical Society of that State.*

On the 19th of September 1802, I was called to visit William M'Cleod, aged 19 years, of whose case I received from his mother the following history, viz. that he had, the evening before, returned from the country, where he had been on a visit to his relations, and had spent with them about a month; that soon after he arose from his bed, this morning, he complained of slight chilliness and a dull pain at the pit of his stomach, which soon after terminated in excessive vomiting, violent fever, and intense pain in his head.

These symptoms continued, without abatement, until about 3 o'clock in the afternoon, when they suffered considerable remission. At this time I saw him. I found that so general a suffusion of bile through the system had taken place, as to resemble a person labouring under jaundice, with the exception of his eyes, which were slightly inflamed.

His mother informed me, that she had taken notice of the yellowness of his skin immediately on his arrival; and on inquiry whether he had been indisposed since his absence, he assured her he had not. His bowels were obstinately bound, having been in a state of constipation for the two or three previous days. His tongue was moist, its edges inflamed, the top white, excepting the middle, down which ran a yellow streak. As his pulse, which was low and irregular, seemed now to forbid the lancet, though there was still some pain in the head, and costiveness and debility appeared to be the principal inconveniences under which he laboured, I contented myself with leaving for him two smart purges of calomel and jalap, with directions to take one immediately, and the other in four hours, if the first did not procure eight or ten copious stools. On visiting him again, about nine o'clock, I found that he had taken both his purges with the happiest effect: they were then operating briskly, and had already produced several large evacuations of hard, dark, and very fœtid fæces. The pain had entirely left his head, his pulse had become regular and more full; a gentle moisture had overspread his skin; his stomach had recovered much of its usual tone, and this was accompanied with a desire for food.

On the morning of the 20th, when I repeated my visit, he had left his bed, with an assurance to his mother, which he he repeated to me, that he felt himself quite free from indisposition. The discharges from his bowels were still kept up; but had entirely lost their fœtor, and appeared to consist chiefly of healthy-looking bile. His skin had become much clearer; as had his urine, which before was of a deep bilious hue. I suggested the propriety of his taking gentle purgatives for a day or two longer; but this advice, from the comfortable state of his feelings, he declined. His mother according with his wishes, I of course left him, and considered my attendance on him as terminated.

About three o'clock P. M. of the 23d, I was again sent for, and desired by the messenger to attend, in all possible haste, as the patient was supposed to be dying. I instantly obeyed the summons, and found his situation such, as to justify the most alarming apprehensions. He was speechless, his jaws were fixed, as were also his eyes, which were nearly closed. He had no pulse at his wrists; near the elbow in one arm I could feel a slight pulsation. His feet, legs and knees, were perfectly cold; and his stools, which were black and very offensive, came from him involuntarily. His breathing had been very laborious, but it now appeared to be free from anxiety. In short, such was the death-like appearance, which his whole countenance exhibited, that I expected every succeeding minute would close his miserable existence. His mother informed me, that the day I left him (the 22d) the pain in his head and fever had returned with its former violence, and had continued without any diminution, until this morning, when it terminated in the comatose state, already described. Having, in repeated instances, witnessed the revival, as it were, and complete restoration of persons from disease, who appeared to be excluded from the smallest ray of hope; I have long determined never to abandon a patient, from motives of despair, in whom I could discover the least signs of life. Under this impression, revolving in my mind, the means I should pursue towards arresting the fatal progress of this case, Mr. Scott's experiments on the nitric acid occurred to me, particularly his opinion in favour of its powers, when "given in sufficient quantity (as he expresses) in acute bilious fevers," and this disorder was obviously of this description. Having determined on the exhibition of this medicine, the only difficulty which now occurred, was the probability of his not being able to swallow; but having determined on the experiment, I diluted the nitric acid to a strength, that I could readily drink myself, and immediately poured a teaspoonful ful into his mouth, whilst his mother forced open his jaws: it lay in his throat at least ten minutes, (during which time, his breathing was marked by that rattling noise, which often takes place in dying persons) when he suddenly swallowed it. On perceiving this, I immediately repeated the dose, which he received without much effort, and before I left him, he had taken a large wine glass full. His mother was now directed to continue the medicine, in as large quantities as his stomach could bear, without any regard to time; and blisters were ordered to be applied to his stomach, wrists, and ancles: perhaps it would have been better to have directed their application to the arms and thighs, as there was more action in those parts; but the propriety of this did not strike me; indeed, I reasoned but little upon them; I only considered them as auxiliaries to the acid, on which I chiefly depended. I visited him again about six o'clock, with little expectation of seeing him alive; but to my astonishment I found him much revived, his eyes had assumed their natural condition, and a slight pulsation was perceptible at his wrists. He still, however, appeared insensible to the objects which surrounded him, and resisted with obstinacy every attempt to give him the acid. During my absence, he had had several involuntary stools, which were very offensive. At 11 o'clock, I repeated my visit. He had taken a measured drachm and a half of the acid, and appeared to be in a sound, undisturbed sleep; the blisters had begun to produce their effects, and warmth was again restored to the extremities. I now recommended the exhibition of the acid every hour, during the night; and in the morning, when I repeated my visit, I was happy to find him so much recovered, as to induce a belief, that the chances of living were, at least, equal to those of dying. He was now sensible, and knew his friends. He still complained much of a pain in his head, to which was directed the application of cloths dipped in cold vinegar. In order to keep up the discharges from his bowels, which were now diminished, I sent him six pills of calomel, of three grains each, with directions to take one every two hours, alternating them with the acid.

This plan was continued through the day, when, in the evening, I found it necessary to assist the pills with a purgative, composed of seven grains of calomel, and fifteen of jalap, which procured him several hard, fœtid stools. 25th, continued the pills and acid: in the evening complained of slight head-ache, pulse tense, skin hot and dry; repeated the cathartic. 26th, much relieved; continue pills pills and acid. 27th, much better; medicines as yesterday; repeated the cathartic. 28th, perceived symptoms of salivation; repeated the pills and acid. 29th. On visiting him this morning I found him out of bed, and walking about the house. His principal complaint now was the state of his mouth; in other respects, he appeared to be quite recovered; and I considered my visits to him no longer necessary. From the 23d to the 30th, he took ten drachms of acid, and ninety-six grains of calomel.

The above case is a plain statement of facts, and as such, I shall leave it, without attempting to offer any observations on the modus operandi of the medicines I employed: for notwithstanding the great number of pages, I might perhaps say, volumes, which have been written on the subject, it is still, in my opinion, involved in much obscurity. But it is an important case, and teaches us never to be discouraged from attempting the relief of our patients, under the most unfavourable appearances: for, as we know not when life begins, so are we, equally, at a loss to know when it will end.

